# A double-edged sword of using opioids and COVID-19: a toxicological view

**DOI:** 10.1186/s13011-020-00333-y

**Published:** 2020-12-03

**Authors:** Mahshid Ataei, Farshad M. Shirazi, Roland J. Lamarine, Samaneh Nakhaee, Omid Mehrpour

**Affiliations:** 1 Medical Toxicology and Drug Abuse Research Center, Birjand University of Medical Sciences, Birjand, Iran; 2 Arizona Poison and Drug Information Center, the University of Arizona, College of Pharmacy and University of Arizona College of Medicine, Tucson, AZ USA; 3 Professor of Public Health, California State University Chico, California, USA; 4 Mel and Enid Zuckerman College of Public Health, University of Arizona, Tucson, AZ USA

**Keywords:** Opioids, Immune system, COVID-19, Prognosis

## Abstract

Today, COVID-19 is spreading around the world. Information about its mechanism, prognostic factors, and management is minimal. COVID-19, as a human disease, has several identifying phases. Physicians of patients with COVID-19 may be interested in knowing whether opioid use disorder may affect their patients’ course or prognosis. This information may be crucial when considering the opioid epidemic in the US and other parts of the world. Opioid use at high doses and over several months duration can mitigate the immune system’s function, which may complicate the course of COVID-19 disease. Potential suppression of parts of the immune response may be important in prevention, clinical support, and therapeutic use of medications in various phases of the COVID-19. Specifically, opioid use disorders via an inhalation route may enhance the “late hyper-inflammatory phase” or result in end-organ damage. It is well established that opioids decrease ventilation as their effect on the medullary respiratory centers increases the risk of pneumonia. This increased risk has been associated with immune-suppressive opioids. The ultimate role of opioids in COVID-19 is not clear. This paper endorses the need for clinical studies to decipher the role and impact of chronic opioid use on viral diseases such as COVID-19.

## Background

Today, COVID-19 is spreading around the world. Information about its mechanisms, prognostic factors, and management is minimal. Like other coronaviruses, SARS-COV2 infects respiratory epithelial cells using receptor-mediated endocytosis via the angiotensin-converting enzyme II (ACE2) as an entry receptor [1, 2]. In symptomatic patients, clinical symptoms usually begin during the first week, this includes fever, fatigue, weakness, cough, nasal congestion, and other signs of upper respiratory tract infection [2]. The infection can eventually lead to serious illness with shortness of breath in approximately 21% of patients and severe pneumonia, as seen by computed tomography scan (CT scan) at admission [3]. Three or four phases for COVID-19 have been potentially identified, including 1- viral replication accompanied by mild symptoms (early infection phase), 2- adaptive immunity stimulation and predominance of respiratory symptoms (pulmonary phase), 3- hyper coagulopathy phase, and in some cases hyper-inflammatory or end-organ damage phase [4]. Before this outbreak, two clinically significant corona virus-associated diseases were known, MERS and SARS, which in most cases, caused infections in patients with adequate immune systems [5, 6]. It is also known that the coronavirus can directly infect macrophages and disrupt T-cell antiviral response by stimulating T-cell apoptosis, thereby disrupting the immune system [7–9]. Theoretically, the immune system status is a crucial factor in managing the virus. Physicians need to know whether, and to what extent, the use of opioids or alcohol may affect patients’ prognosis with COVID-19. This information will be especially critical when considering the opioid epidemic in the US and other parts of the world.

COVID-19 and opioid use disorder are two syndemics that pose a significant threat to public health. Some articles’ primary focus is on Opioids and COVID-19, and the main narrative relates to opioid prescription, opioid access, and toxicological considerations [10, 11]. Epidemiologic studies have shown an increase in mortality rate in people with opioid use disorder with COVID-19 during the pandemic. For example, Wang et al. demonstrated that people with substance use disorder, particularly recent opioid use disorders, are at higher risk for COVID-19 (adjusted odds ratio = 10.244 [9.10–11.52], *P* < 10^− 30^) and worse outcomes (death: 9.6%, hospitalization: 41.0%, *P* < 0.05) [12].

Immune suppression in people with opioid use disorder has been suspected for many years. Some data indicate an increase in infection incidence in people with opioid use disorders [13]. According to previous studies, opioids have a complex effect on the immune system. Some opioids, such as morphine and fentanyl, seem to cause immune suppression and increase the risk of pneumonia; others, such as buprenorphine and oxycodone, may enhance the immune response [14]. Low doses and short-term, temporary use of some opioids may stimulate immune cells and the body’s defense [14–17]. However, it is clinically evident that people with opioid use disorder and chronic use are at higher risk of infections, specifically respiratory illnesses. This complexity of interaction is not only opioid-dependent but also depends on its duration [17]. Our goal is to stimulate conversation and interest in research on potential interactions and modulation of the immune system with opioids in patients with COVID − 19 disease.

## Main body

The opioids modulation of the immune system can both exacerbate and potentiate the therapeutic effects in the treatment of covid-19 disease. This modulation of the immune system and its therapeutic potential can also be affected by the chronicity of use of this class of agents.

A systematic in vitro study has shown that opioids such as morphine suppress the cellular immune system and response to bacterial infection; this finding has been validated by epidemiological studies [18].

In contrast, codeine, oxycodone, diamorphine, and methadone did not produce a measurable effect on the immune system [19]. Another related study indicated that morphine leads to the induction of apoptosis in the immune cells, atrophy of the thymus and spleen, and suppression of proliferation of B and T lymphocytes [20]. Other studies have reported opium as an immunosuppressive agent and causes diminished leucocyte activity by inhibiting the bone marrow’s migration. This effect can be offset by injection of naltrexone [21]. In vitro studies and animal models cannot provide conclusive evidence for opioids’ impact on the human immune system. Although there is no direct clinical evidence for the detrimental impact of opioids, epidemiologic studies tend to validate opioid effects on immune suppression and exacerbation of infection in respiratory illnesses [22]. A case-control study in 2007 by Azarang et al. showed that opium caused a diminished mitogenic response of lymphocytes [23]. In a review by Wang et al. in 2008, it was noted that opiates such as morphine impair the first defense line of the body against bacteria by reducing macrophages, neutrophils, nonspecific cytotoxic T cells, natural killer cells, and dendritic cells, thus increasing the risk of bacterial infections [24]. Some studies suggest that certain drugs that act as μ-receptor agonists tend to cause immunosuppression, while antagonists tend to enhance or have little effect on immune function [25]. Opioid-induced immune modulation has long been thought to occur through direct actions on the immune cell itself or via the hypothalamic-pituitary-adrenal (HPA) axis, or combination of both mechanisms. One study reported that opioid receptor mRNA (except NOP (nociceptin / orphanin FQ receptor, which is not sensitive to naloxone)) was not found on circulating immune cells. The evidence for HPA activation is also insufficient and shows some species dependence [26].

Overall, it can be surmised that opioid use at high doses and over several months would suppress immune system function that could worsen the prognosis for the organ phase of COVID-19, which results in endothelium damage of lung’s alveolar and co-infection with bacterial pneumonia.

Opioids have respiratory depressant effects [27], and many people with opioid use disorder may administer the opioids via an inhalation route and may be more susceptible to infections such as SARS-COV2. Besides, it is well known that opioids may decrease ventilation, probably by inducing a decrease in the medullary respiratory centers’ sensitivity and responsiveness to hypoxia and hypercapnia [28], worsening the respiratory phase of COVID-19 [1].

Additionally, patients with opioid use disorder due to various socio-economic factors such as lack of access to health care and supportive services, housing instability, poor health condition, and congregate opioid use may not abide by social distancing and other preventive measures. The conditions mentioned above will increase this population’s susceptibility to infection with SARS-COV2 [29]. Therefore, further research should be done on socio-economic conditions that increase infection risk and potential immune modulation due to opioids use in this population.

Although immune suppression caused by opioids may facilitate the entrance of viruses into the body and have adverse effects that may increase comorbidity, recent studies have shown that inflammatory cascade and cytokine storm syndromes are evident in some disease phases and may worsen these patients’ prognosis. In this regard, the cytokine storm has been documented in most severe COVID-19 patients [30]. High levels of various cytokines such as IL-2, IL-7, IL-,10, and TNF-α were measured in the serum of critically ill cases of COVID-19 [30]. The high concentration of cytokines may be considered as a marker of severity in these patients [3]. Hyper-inflammatory syndrome may lead to multi-organ failure and death [3, 31]. So, immune suppression, which decelerates the inflammatory process, might play a protective role in some COVID-19 patients [32]. In this regard, previous studies have shown that Janus kinases (jacks) have a vital role in cytokine signaling, so inhibition of Jak activity may be a promising strategy for suppressing the immune system [33]. JAK inhibitors such as baricitinib have been reported to affect inflammation and cellular viral entry and suppress Interleukins in COVID-19 [34]. It has also been argued that acute use of opioids such as morphine also competes with binding of SARS-COV2 to the angiotensin II receptors and suppresses interleukin levels [35]. These authors have gone so far as to suggest a protective role for such opioids. However, the exact effect of chronic use of the same opioids on angiotensin II binding and interleukin levels has not been well established. What is clear is that patients with opioid use disorder are at higher risk of contracting and developing severe COVID-19 disease [12].

Although the significant overlap between the different phases of COVID-19 may occur in individual patients, the effects of opioids or even the proposed treatments may differ at each stage. For example, the use of antiviral agents to limit the spread of the virus is more helpful if used in the early phases, but the use of immunosuppressive regimens, such as anti-interleukin (IL) -6 such as Tocilizumab has been shown to have some beneficial role in patients with COVID-19 pneumonia and increased IL-6 [36]; also there are some benefits of using anti-interleukin (IL)-1 or corticosteroids, in the second and third phases. Various opioids in chronic use appear to have different effects in suppressing the immune system and modulating the cytokine release. Depending on the type of opioid and the chronicity of use, people with opioid use disorder may respond differently to infection with SARS-COV2 at different phases of COVID-19 disease. Clinically speaking, opioid use disorder may increase the disease’s complications and severity or modulate cytokine storms and be beneficial. However, epidemiological evidence, so far available, indicates a higher susceptibility of patients with opioid use disorder in contracting COVID-19 disease and having more severe and detrimental effects [12]. At this time, it is hard to distinguish whether it is the comorbidities or the immunological impact of opioids that are responsible for a more severe course of COVID-19 in patients with opioid use disorder. We suggest that even controlling for socio-economic conditions, the patients with opioid use disorder will have a worse outcome due to the physiological and immunological impact of opioids.

As mentioned earlier, studies in people with opioid use disorder have demonstrated thatopioids consistently can cause immunosuppression, includingsignificant cellular immunity impairment andT cell genetic damage [37]. Furthermore, treatment of opioid use disorder with buprenorphine, methadone, naltrexone may also regulate the immune response. For example, cellular immunity suppression in patients with opioid use disorder can return to normal by switching to long-term methadone treatment [37]. Accordingly, a randomized clinical trial reported that both methadone and partial mu agonists, such as buprenorphine, could activate the immune systems in people who use heroin [38]. A recent study that evaluated the risks and outcomes for COVID-19 in people with substance use disorders (SUD) indicates that medications used to treat opioid use disorder (OUD) had no significant effects on patients’ risk for COVID-19 after adjusting for age, gender, race, and insurance type [12].

During the pandemic, it has also been noted that lack of access to detox and treatment combined with social isolation increases one’s vulnerability to relapse and overdose.

## Conclusion

Opioid use at high doses and over several months could suppress immune system function and affect the prognosis for cases of COVID-19. However, it has been suggested that immunosuppression might play a protective role during COVID-19 infection. Clinical studies should evaluate various aspects of using opioids and the prognosis of patients who use opioids and have COVID-19 infection. Innovative public health policies must be widely discussed, evaluated, and implemented in order to overcome these disparities. Also, the patients using chronic opioids therapeutically or those misusing opioids should be considered a clinically vulnerable group for careful monitoring.

## Data Availability

Data used to make the conclusion are provided in this letter. Any conclusions drawn in this letter to the editor reflect the authors’ points of view and not the Institution of Affiliation.

## Abbreviations

COVID-19: Corona Virus disease 2019
ACE 2: Angiotensin-converting enzyme II
US: United States of America
MERS: Middle East respiratory syndrome coronavirus
SARS: Severe acute respiratory syndrome-related coronavirus
CT scan: Computed tomography scan
OP3: Mu opioid receptors
